# Illegitimate Tasks and Work Motivation: Examining the Full Continuum of Self‐Determination

**DOI:** 10.1111/sjop.70025

**Published:** 2025-09-08

**Authors:** Petri Karkkola

**Affiliations:** ^1^ School of Educational Sciences and Psychology University of Eastern Finland Joensuu Finland

**Keywords:** amotivation, illegitimate tasks, intrinsic motivation, self‐determination, work motivation

## Abstract

Illegitimate tasks are tasks that are perceived as unnecessary or unreasonable. They act as stressors and are expected to induce various strains on employees, including motivational strains. In previous studies, only the association between illegitimate tasks and intrinsic motivation was examined. In the present three‐wave longitudinal study, the examination was expanded to include the full motivational continuum described in self‐determination theory. Structural equation modeling was used to examine both cross‐sectional and longitudinal associations between illegitimate tasks and behavioral regulations among Finnish working adults. In addition to intrinsic motivation, illegitimate tasks were observed to also be strongly associated with amotivation and more moderately associated with external regulation, introjected regulation, and identified regulation in the expected directions. In longitudinal analyses, illegitimate tasks were observed to predict an increase in amotivation and a decrease in autonomous motivation; introjected regulation was observed to predict an increase in illegitimate tasks; and autonomous motivation was observed to predict a decrease in illegitimate tasks. Illegitimate tasks and motivation described in self‐determination theory may have reciprocal associations over time. Examining other behavioral regulatory styles in addition to intrinsic motivation is recommended.

**Summary**

- The study examines cross‐sectional and longitudinal associations between illegitimate tasks and behavioral regulatory styles depicted in seld‐determination theory.
- In cross‐sectional analyses, illegitimate tasks were most strongly associated with intrinsic motivation and amotivation, and more moderately associated with other regulatory styles.
- Longitudinal analyses suggested partly reciprocal associations: illegitimate tasks predicted higher amotivation and a lower autonomous motivation, and autonomous motivation predicted fewer illegitimate tasks.

## 1 Introduction

Illegitimate tasks are defined as tasks that employees perceive to violate expectations about what can reasonably be expected of them (Semmer et al. 2010). Tasks can be appraised as illegitimate because they are seen as unreasonable or unnecessary. Unreasonable tasks are regarded as being outside of the range of an employee's occupation due to inappropriate content, restrictions, or skill demands. Unnecessary tasks, on the other hand, are tasks that are perceived as tasks that should not exist because they do not make sense or could be avoided by organizing work better (Semmer et al. 2010). Illegitimate tasks are often operationalized as one factor of experienced illegitimate tasks, but studies have suggested that the facets of unreasonable tasks and unnecessary tasks may sometimes be differentially associated with relevant outcomes or antecedents. For example, perceived unreasonable tasks may be more strongly associated with negative emotions (Pindek et al. 2019) and may affect counterproductive work behavior more consistently than unnecessary tasks (Schulte‐Braucks et al. 2019). Specifically, a high level of unnecessary tasks has been observed to be associated with a low subsequent meaning of work, although changes in both facets predicted changes in the meaning of work (Mäkikangas et al. 2023).

According to the Stress as Offense to Self (SOS) theory, illegitimate tasks can threaten self‐esteem and professional identity through self‐evaluation and evaluation by others (Semmer et al. 2019). Self‐evaluation contributes to stress through insufficiency (e.g., the employee appraises their own performance that they can affect as inadequate), and evaluation by others is a stressor through disrespect (e.g., in social interactions and task assignments). Altogether, illegitimate tasks can be considered stressors that can lead to many negative outcomes via negative emotions, role stress, perceived injustice, and psychological and physiological strain (Ding and Kuvaas 2023).

Illegitimate tasks can be expected to relate to work motivation. Indeed, experiencing illegitimate tasks has been associated with turnover intentions (Apostel et al. 2018; Cregård and Corin 2019; Zeng et al. 2021) and several forms of counterproductive work behavior (Schulte‐Braucks et al. 2019; Semmer et al. 2010; Zhao et al. 2022). Illegitimate tasks may hinder the motivational potential of job demands (Kronenwett and Rigotti 2019) and are associated with supervisor‐rated task performance via job identity (Ma and Peng 2019). They are also associated with lower meaningfulness of work both concurrently (Kilponen et al. 2021) and longitudinally (Mäkikangas et al. 2023) and with lower living a calling at work (Mauno et al. 2022). Only a few studies of illegitimate tasks have focused on their association and dynamics with directly reported experiences of work motivation, and in those studies, only intrinsic motivation has been examined (Fila et al. 2023; Muntz and Dormann 2020; Omansky et al. 2016). In this study, I aim to provide a more comprehensive examination of the relationship between illegitimate tasks and work motivation as described in self‐determination theory (Ryan and Deci 2002, 2017). The study contributes to knowledge concerning associations of illegitimate tasks with both more self‐determined and less self‐determined experiences of work motivation and their temporal dynamics.

### 1.1 Self‐Determination Theory and Illegitimate Tasks

SDT is a macro‐theory of motivation, well‐being, and growth. SDT posits that all people have the basic psychological needs of autonomy, competence, and relatedness, and these needs must be satisfied to promote one's psychological health and development (Ryan and Deci 2017). The need for autonomy is satisfied when a person is experiencing volition and has a sense of ownership of their actions. The need for competence is satisfied when a person perceives themselves as an effective agent in their environment and feels that they have the capacity for development. The need for relatedness is satisfied when a person has positive and reciprocal relationships with individuals and groups in their environment (see, e.g., Ryan and Deci 2002, 2017; Vansteenkiste et al. 2023). In this study, I focus on the motivational qualities assumed to be consequences of the satisfaction of basic psychological needs, called behavioral regulatory styles (Ryan and Deci 2017; Pelletier and Rocchi 2023).

Behavioral regulatory styles concern the perceived reasons to engage in behaviors such as work (Pelletier and Rocchi 2023). Some reasons are considered more extrinsic (such as pay or status) and some more intrinsic or volitional (such as interest and joy). Regulatory styles can be thought to form a continuum in the dimension of self‐determination (Howard et al. 2017), meaning that they can be arranged from the least self‐determined (amotivation) to the most self‐determined (intrinsic motivation). Satisfaction of the basic psychological needs is assumed to foster more self‐determined regulatory styles, whereas deficits in need satisfaction are thought to lead to less self‐determined styles (Ryan and Deci 2017).

In the least self‐determined end of the continuum, amotivation refers to a state in which there is no motivation to act or behavior is not regulated by intentions (Ryan and Deci 2017). According to Ryan and Deci (2017), this non‐intentionality can be brought about by the perceived inability to attain an outcome, the low utility of the outcome, or the absence of interest in behavior. In the work context, amotivation has been associated especially with higher burnout symptoms, lower performance, more frequent counterproductive work behavior, and lower job satisfaction (Van den Broeck et al. 2021).

External regulation describes motivation dependent on external contingencies, such as external pressures, rewards, and punishments (Ryan and Deci 2017). It is sometimes divided into external social regulation and external material regulation (Gagné et al. 2015), but they have mostly been examined as a single dimension (Van den Broeck et al. 2021). At work, external regulation has been especially associated with higher continuance commitment (Van den Broeck et al. 2021).

Introjected regulation requires internalization because it is based on affective and evaluative contingencies within an individual, unlike the previously mentioned regulations (Ryan and Deci 2017). However, it makes self‐worth contingent on behaviors or characteristics that are only partially internalized (Pelletier and Rocchi 2023). The experience of introjected regulation may comprise pride and guilt due to its non‐self‐determined nature (Ryan and Deci 2017). Perhaps reflecting its tense character, introjected regulation has been positively associated with both burnout symptoms and work engagement, and with both affective commitment and normative commitment (Van den Broeck et al. 2021).

When acting out of identified regulation, the employee has better internalized the value or personal importance of the activity, albeit not completely (Ryan and Deci 2017). Identified regulation loosens the connection between an activity and external demands (Pelletier and Rocchi 2023). Nevertheless, the different identifications are not necessarily fully congruent. Ryan and Deci (2017) call this compartmentalization of identifications, in which some identifications remain actively and defensively unintegrated. In the work context, identified regulation is consistently positively associated with well‐being, performance, and organizational citizenship behavior and negatively associated with ill‐being (Van den Broeck et al. 2021).

Integrated regulation refers to experiencing perfect alignment of one's behavior with one's goals and identity (Pelletier and Rocchi 2023). According to Ryan and Deci (2017), an active transformational process and self‐reflection are often required to achieve full congruence among different aspects of the self. It is difficult to distinguish integrated regulation from identified regulation empirically (Howard et al. 2017), and their associations with work‐related phenomena are virtually identical (Van den Broeck et al. 2021). The work motivation measure used in this study omits integrated regulatory style, and therefore, the style is not used in the analyses.

Finally, intrinsic motivation refers to engaging in activities because they are inherently enjoyable and interesting (Ryan and Deci 2017). Behavior does not require external or internal controls. Intrinsic motivation at work is associated quite strongly with higher well‐being and affective commitment and more moderately with higher performance, proactivity, and more frequent organizational citizenship behavior (Van den Broeck et al. 2021).

To date, all previous studies that have examined illegitimate tasks and work motivation as described in SDT have focused on intrinsic motivation. In cross‐sectional studies, associations between illegitimate tasks and lower intrinsic motivation have been observed (*r* ≤ −0.31; Fila et al. 2023; Omansky et al. 2016). Additionally, Fila et al. (2023) observed that the effect of illegitimate tasks on turnover intention and strain was stronger if intrinsic motivation was high (rather than low). This was interpreted to suggest that intrinsic motivation can act as a vulnerability factor if stressors such as illegitimate tasks hinder the confirmation of one's professional identity.

Using two measurement points across two weeks, Muntz and Dormann (2020) found that the correlations between illegitimate tasks from different sources (supervisors, colleagues and patients) and intrinsic motivation were small and mostly statistically nonsignificant (*r* ≤ 0.11). They also tested the temporal relationship between illegitimate tasks and intrinsic motivation, concluding that it is more probable that illegitimate tasks, specifically unnecessary tasks, are the consequence of intrinsic motivation. For example, intrinsic motivation increased the perceived unnecessary tasks assigned by colleagues. These results may be partly explained by the methodological choices made. Muntz and Dormann (2020) used the intrinsic motivation measure devised by Warr et al. (1979), whose contents do not necessarily align with SDT's conception of intrinsic motivation. Most items of the measure contain some contingency statements regarding performing well (“My opinion of myself goes down when I do this job badly”; “I take pride in doing my job as well as I can”), which better correspond to the definition of introjected regulation. The items do not include statements of sheer enjoyment and interest in the job as such.

Due to their definition as tasks that are perceived as unreasonable or unnecessary, illegitimate tasks themselves do not foster self‐determined behavior regulation. Indeed, a more important question is why or how the perception of the presence of illegitimate tasks is associated with concurrent or future work motivation. Muntz and Dormann (2020) analyzed the conceptual relationship between illegitimate tasks and basic psychological needs in SDT. They stated that illegitimate tasks hinder autonomy satisfaction because they are always assigned by others and are carried out involuntarily and contradict competence because they require skills above or below an employee's qualifications (Muntz and Dormann 2020). They also posited that illegitimate tasks threaten relatedness because they convey a sense of not being accepted by others. It can be speculated that in addition to being inherently demotivating, illegitimate tasks threaten one's work motivation as a whole through stress from insufficiency (e.g., appraising one's own performance as inadequate) and stress from evaluation by others (e.g., assigning illegitimate tasks may signal disrespect; Semmer et al. 2019). These processes are detrimental especially to the satisfaction of competence and relatedness. Additionally, illegitimate tasks may compete with tasks that the employee perceives as more relevant to one's professional identity, which may hinder autonomy satisfaction.

Because satisfaction of basic psychological needs has a major role in determining motivational experiences (Ryan and Deci 2017; Ryan and Vansteenkiste 2023; Pelletier and Rocchi 2023; Van den Broeck et al. 2016, 2021), it can be expected that, in general, illegitimate tasks are most strongly associated with the regulatory styles located in the extreme ends of the self‐determination continuum and not as strongly with the regulatory styles in the middle. Therefore, illegitimate tasks should be associated most strongly with low intrinsic motivation, low integrated regulation (two most self‐determined motivational experiences that also correlate highly), and high amotivation (the least self‐determined regulatory style).

### 1.2 Research Questions and Hypotheses for the Present Study

In this study, I examine the associations between perceived illegitimate tasks and the full continuum of behavioral regulatory styles at work according to SDT. This significantly contributes to the literature by providing a more comprehensive account of the phenomena in question, compared to the previous studies limited to intrinsic motivation only. By examining the regulatory styles simultaneously, the specific associations of each style with illegitimate tasks can be observed. Furthermore, the longitudinal design of this study facilitates the understanding of the temporal and potentially causal relationships of illegitimate tasks and regulatory styles. From the point of view of SDT and SOS, illegitimate tasks can be considered stressors that cause negative emotional and motivational effects. However, in the only longitudinal study so far, the observed predictive relations were small and suggested some of the perceived illegitimate tasks to be a consequence of high intrinsic motivation (Muntz and Dormann 2020). This observation is surprising regarding both the temporal relation and the direction of the association. This study substantively clarifies the roles of the examined factors by utilizing proper SDT‐based measures in a longitudinal design.

The research questions and corresponding hypotheses in the present study are as follows:

1. What are the cross‐sectional associations between illegitimate tasks and regulatory styles? The hypotheses are based on the general idea that the associations are dependent on the regulatory style's position on the self‐determination continuum. According to Hypothesis 1a, illegitimate tasks are associated with low intrinsic motivation with an effect size of approximately *r* ≈ −0.30, a magnitude based on previous studies (Fila et al. 2023; Omansky et al. 2016). According to Hypothesis 1b, illegitimate tasks are associated with low identified regulation with a somewhat lower effect size (*r* ≈ −0.20). According to Hypothesis 1c, the association between illegitimate tasks and introjected regulation is small but positive (*r* ≈ 0.10). According to Hypothesis 1d, associations between illegitimate tasks and external regulatory styles (both material and social regulation) are positive, with a medium of approximately *r* ≈ 0.20. Finally, according to Hypothesis 1e, illegitimate tasks are associated with amotivation with an effect size comparable to that of illegitimate tasks and intrinsic motivation but with a different sign (*r* ≈ 0.30).
2. What is the temporal association of illegitimate tasks and regulatory styles? According to Hypothesis 2, based on the SDT model in the workplace (Deci et al. 2017), perceived illegitimate tasks are considered stressors that are expected to predict especially low intrinsic motivation and high amotivation.

## 2 Materials and Methods

### 2.1 Participants and Procedure

Working adults (at least 18 years old) who were fluent in Finnish were invited to participate in this study via a market research company. Participants received a small fee. Those who agreed to participate in the study provided their informed consent to participate in it, after which they completed the study questionnaire online. The study protocol was approved by the ethical review board of the author's university.

The data were collected at three measurement points in 2022, with intervals of about four months. Of the 822 respondents at Time 1 (T1), 706 were deemed careful responders (see the Statistical analyses section) and were included in the sample. At Time 2 (T2), there were 462 careful responders, and at Time 3 (T3), there were 411, 65% and 58% of the originally included participants, respectively. Assessment of careful responding was conducted after the data collection, and every participant who responded at T1 received the invitation to participate in both subsequent measurement points.

### 2.2 Measures

Illegitimate tasks were assessed using the Bern Illegitimate Tasks Scale (BITS; Semmer et al. 2015). It includes a stem (“Do you have work tasks to take care of which keep you wondering if…”) and four items for unnecessary tasks (e.g., “…they have to be done at all”), and stem “Do you have work tasks to take care of, which you believe… “and four items for unreasonable tasks (e.g., “…are unfair that you have to deal with them”). The items were rated by the respondents on a scale from 1 (“never”) to 5 (“frequently”).

Regulatory styles were assessed with the Multidimensional Work Motivation Scale (MWMS; Gagné et al. 2015). It is a 19‐item measure and includes a stem about reasons to put effort into one's work and three items for amotivation (e.g., “I don't know why I'm doing this job, it's pointless work”), three items for external material (e.g., “Because I risk losing my job if I don't put enough effort in it”), three items for external social regulation (e.g., “To get others' approval”), four items for introjected regulation (e.g., “Because otherwise I feel bad about myself”), three items for identified regulation (e.g., “Because putting efforts in this job aligns with my personal values”), and three items for intrinsic motivation (e.g., “Because the work I do is interesting”). MSWM omits integrated regulation. The items are responded to on a scale from 1 (“not at all”) to 7 (“completely”).

Information on participants' age (in years), gender (man/woman/other), and work contract (full time or part time and fixed or non‐fixed term) was collected for descriptive purposes. Age and gender were used also as control variables in longitudinal analyses.

### 2.3 Statistical Analyses

Attrition analyses were conducted using IBM SPSS Statistics (Version 28). Prior to other analyses, careless responding was examined, such as inconsistent responses, intraindividual response variability (Dunn et al. 2018), responding without considering the item content (statistical synonyms; Meade and Craig 2012) and repeating single responses (with LongString macro for Microsoft Excel; Landers 2020). Responders deemed careless at the first measurement point (*n* = 116) were removed from the data, and careless respondents at the latter measurement points (*n* = 46 at T2, *n* = 28 at T3) were considered missing data. The participants who dropped out after T1 were compared with the other participants. Comparisons were made with a *t*‐test or Pearson *χ* ^2^ test, depending on the variable, and *p* < 0.01 was used as a criterion for a significant difference between the responders and non‐responders. The results were depicted as either percentages or standardized mean differences (Cohen's *d*).

The research questions were examined using structural equation modeling (SEM) with latent variables, with robust maximum likelihood estimation in Mplus (Version 8.9). Missing data were processed with the default full information maximum likelihood estimation. In the measurement models and further structural models, both the comparative fit index (CFI) and Tucker–Lewis index (TLI) were considered to indicate satisfactory (≥ 0.90) or good fit (≥ 0.95), and both root mean square error of approximation (RMSEA) and standardized root mean square residual (SRMR) were considered to indicate satisfactory (≤ 0.08) or good fit (≤ 0.05; for the criteria, see Little 2013).

Motivation and related behavioral regulatory styles have been conceptualized and operationalized in various ways (see, e.g., Bureau et al. 2023; Howard et al. 2020; Trépanier et al. 2023). For MWMS, four longitudinal measurement models were tested for configural, metric, and scalar invariance. First, a six‐factor model with factors for amotivation, external material regulation, external social regulation, introjected regulation, identified regulation, and intrinsic motivation was tested. Subsequently, a five‐factor model otherwise identical to the previous model but with identified regulation and intrinsic motivation as one factor of autonomous motivation was tested. A four‐factor model with the external material regulatory style, external social regulatory style, and introjected regulatory style as a factor of controlled motivation was also tested. Finally, a three‐factor model with amotivation, controlled motivation, and autonomous motivation was tested.

Regarding the first research question (cross‐sectional associations between illegitimate tasks and regulatory styles), the objective was to identify as well‐fitting a model of MWMS as possible with as many theoretically justified regulatory style factors as possible. This approach was taken for two reasons. First, each regulatory style in SDT has a clear theoretical definition and a specific location on the self‐determination continuum. Therefore, their associations with illegitimate tasks can potentially provide novel insights into the nature of experienced task illegitimacy. For instance, although the most self‐determined regulatory styles are empirically strongly associated (e.g., Howard et al. 2017), intrinsic motivation is conceptually distinct from the relatively autonomous identified and integrated regulatory styles. Intrinsic motivation is based solely on the interest in and enjoyment of the activity itself, rather than any external identifications or valuations. Secondly, strong associations between variables are not a threat to the validity of results in correlational analyses (cf. multicollinearity in regression analysis). For similar reasons, illegitimate tasks were modeled by two distinct but related factors of unnecessary tasks and unreasonable tasks that were added to the chosen MWMS model, and the whole model was tested for configural, metric, and scalar invariance. Although differences in associations between regulatory styles and the two facets of illegitimate tasks were not hypothesized, modeling them both allows for observing potential differences, provided that the measurement model with two illegitimate task factors is psychometrically sound.

For the second research question (temporal relations of illegitimate tasks and regulatory styles), the aim was to identify a similarly maximally informative model of MWMS and BITS without excessive multicollinearity. First, the MWMS measurement models tested above were inspected regarding model fit and factor correlations, and a model with both adequate fit and low multicollinearity was chosen for further testing. Next, two models with illegitimate tasks were tested. In the first model, illegitimate tasks were modeled with two latent factors as described above. In the second model, illegitimate tasks were modeled with one latent factor, whose indicators were the observed scales of unnecessary tasks and unreasonable tasks (Semmer et al. 2015). Models were tested for configural, metric, and scalar invariance, and a model for cross‐lagged analyses was selected based on fit statistics and multicollinearity between factors of illegitimate tasks.

Exploratory structural equation modeling (ESEM; Marsh et al. 2014) was utilized because it corresponds with the theoretical framework of and empirical results regarding the dimensionality of motivation in SDT (Howard 2023; Howard et al. 2017, 2018; Trépanier et al. 2023). The MWMS items were rotated with target rotation, allowing cross‐loading on all the motivation factors but attempting to minimize them, while the BITS items remained unrotated and were loaded only on their respective factors. For graphical representations of different kinds of models, see Howard et al. 2020, Image 1.

In the testing of the measurement models, measurement invariance was deemed to be supported if the change in the chi‐squared value between the successive models was statistically insignificant (*p* ≥ 0.05) or if the change was statistically significant but CFI decreased by less than 0.010 and RMSEA increased by less than 0.015 (for the criteria, see Chen 2007). The scalarly invariant measurement model was re‐specified as an ESEM‐within‐CFA model to allow for the use of the models utilizing regression analyses (Marsh et al. 2014; Morin et al. 2013).

Regarding the first research question, three successive models were compared to obtain an appropriately parsimonious model for the associations. First, the scalarly invariant measurement model was compared to a model in which covariances between illegitimate tasks and regulatory styles were fixed to be equal across measurement points, because the associations were not expected to differ in strength due to the measurement occasion (e.g., the association between unnecessary tasks and intrinsic motivation was assumed to equal at T1, T2, and T3). Second, the model with equal covariances across measurement points was compared to a model in which also the covariances of both factors of illegitimate tasks were fixed to be equal with each respective regulatory style (e.g., in addition to the restrictions above, the association between unnecessary tasks and intrinsic motivation was assumed to be equal with the association between unreasonable tasks and intrinsic motivation). There were no theoretically compelling reasons to expect that the two aspects of illegitimate tasks would be associated with behavioral regulations in different ways, but comparing the models allowed for explicit testing of the potential differences. The more parsimonious model was deemed to be supported if the change in the chi‐squared value between the successive models was statistically insignificant (*p* ≥ 0.05) or if the change was statistically significant, but CFI decreased by less than 0.010 and RMSEA increased by less than 0.015. Correlations and their 95% confidence intervals (CIs) of the selected model are reported. Although the covariances are fixed to be equal, correlations may slightly differ.

For the second research question, an autoregressive model was specified, each factor freely predicting the corresponding factor at the next measurement point. Factor correlations were estimated at T1 and residual factor correlations were estimated at T2 and T3 between all factors. Correlated uniqueness of the indicators was estimated over time for each indicator. Next, an autoregressive model with predictive equilibrium (predictive paths constrained to be equal across measurement points) was specified and compared to the previous autoregressive model. The equilibrium model was retained, provided that the change was statistically insignificant (*p* ≥ 0.05) or if the change was statistically significant but CFI decreased by less than 0.010 and RMSEA increased by less than 0.015. Next, cross‐lagged panel models with freely estimated predictive paths were specified and compared to the retained autoregressive model. The cross‐lagged models were deemed better than the autoregressive model if the change in the chi‐squared value between the models was statistically significant (*p* < 0.05). Provided that the cross‐lagged panel model fitted the data better compared to the autoregressive model, constrained predictive paths were added to the cross‐lagged model, making it an equilibrium model. This model was compared to the model with freely estimated cross‐lagged paths. For a similar procedure with freely estimated and constrained predictive paths, see Garn et al. (2019).

Three cross‐lagged models were tested. The first model, an illegitimate tasks to behavioral regulations model, suggested that illegitimate tasks predict the regulatory styles at the next measurement point. The second model, behavioral regulations to illegitimate tasks model, suggested that regulatory styles predict illegitimate tasks. Finally, the third model, reciprocal model, suggested that illegitimate tasks predict the regulatory styles, and the regulatory styles predict illegitimate tasks. In addition to fit statistics, standardized regression coefficients (*β*) and their statistical significance were inspected. Regardless of the model, one‐tailed significance testing was utilized, assuming that all autoregressive associations are positive and that among cross‐lagged associations, illegitimate tasks are associated positively with amotivation, external regulatory styles (both material and social) and introjected regulation, and negatively with identified regulation and intrinsic motivation. All models were adjusted for age and gender. Due to their small number, the gender information of non‐binary responders had to be processed as missing data to avoid estimation problems and to keep their responses in the analyses.

## 3 Results

### 3.1 Descriptive Statistics

Depending on the measurement point, the participants' mean age was 40.50 to 42.34 (standard deviation: 11.03–12.16). The participants mostly identified as men (44%–46%) or women (54%–56%), with nonbinary persons accounting for only < 1% of the sample at all the measurement points. Most of the participants (75%–78%) worked full‐time and with non‐fixed terms (74%–81%).

The participants who opted out after T1 were younger (36.1 years) than those who did not (42.2 years; *t*_704_ = 6.28, *p* < 0.001), and they experienced stronger amotivation (*t*_704_ = −5.29, *p* < 0.001, *d* = −0.44) and more extrinsic material regulation (*t*_704_ = −3.22, *p* < 0.001, *d* = −0.27) at T1. Attrition among the participants with fixed‐term employment was greater (52%) than that among the participants with non‐fixed‐term employment (24%; *χ* ^2^ = 14.96, *p* < 0.001). The scale scores, reliabilities, and correlations of the study questionnaire, based on the composite variables, are shown in Table 1.

**TABLE 1:** Descriptive statistics of the study scales.

	M (SD)	*ω*	1	2	3	4	5	6	7	8	9	10	11	12	13	14	15	16	17	18	19	20	21	22	23
T1																									
1. AM	2.24 (1.52)	0.94	1																						
2. EMR	3.61 (1.48)	0.78	0.29***	1																					
3. ESR	3.86 (1.39)	0.87	0.17***	0.51***	1																				
4. INR	3.92 (1.40)	0.81	0.10**	0.47***	0.54***	1																			
5. IDR	4.51 (1.49)	0.89	−0.22***	0.20***	0.30***	0.54***	1																		
6. IM	4.64 (1.48)	0.93	−0.28***	0.11**	0.16***	0.24***	0.68***	1																	
7. UN	2.92 (0.92)	0.87	0.44***	0.17***	0.18***	0.17***	−0.11**	−0.26***	1																
8. UR	2.64 (0.89)	0.86	0.44***	0.19***	0.23***	0.20***	−0.06	−0.16***	0.61***	1															
T2																									
9. AM	2.17 (1.50)	0.95	0.68***	0.22***	0.06	−0.00	−0.30***	−0.37***	0.35***	0.34***	1														
10. EMR	3.52 (1.45)	0.78	0.23***	0.61***	0.36***	0.41***	0.18***	0.10*	0.10*	0.13**	0.23***	1													
11. ESR	3.83 (1.37)	0.87	0.06	0.34***	0.58***	0.43***	0.21***	0.06	0.16***	0.21***	0.07	0.45***	1												
12. INR	3.98 (1.43)	0.84	−0.08	0.31***	0.47***	0.64***	0.43***	0.24***	0.10*	0.05	−0.04	0.43***	0.56***	1											
13. IDR	4.53 (1.50)	0.91	−0.30***	0.07	0.19***	0.38***	0.67***	0.60***	−0.15***	−0.13**	−0.33***	0.21***	0.26***	0.54***	1										
14. IM	4.53 (1.53)	0.94	−0.31***	0.05	0.11*	0.24***	0.55***	0.76***	−0.28***	−0.24***	−0.40***	0.13**	0.10*	0.36***	0.75***	1									
15. UN	2.95 (0.92)	0.89	0.33***	0.00	0.06	0.05	−0.16***	−0.34***	0.65***	0.46***	0.41***	0.07	0.13**	0.06	−0.18***	−0.33***	1								
16. UR	2.62 (0.84)	0.87	0.34***	0.05	0.14**	0.06	−0.13**	−0.23***	0.49***	0.62***	0.37***	0.12*	0.17***	0.06	–0.09	−0.23***	0.66***	1							
T3																									
17. AM	2.03 (1.32)	0.93	0.59***	0.23***	0.10*	0.02	−0.26***	−0.33***	0.36***	0.31***	0.60***	0.16**	0.08	−0.06	−0.35***	−0.38***	0.33***	0.25***	1						
18. EMR	3.51 (1.40)	0.76	0.23***	0.64***	0.30***	0.34***	0.16***	0.11*	0.07	0.12*	0.14**	0.64***	0.35***	0.36***	0.18***	0.16**	0.02	−0.07	0.17***	1					
19. ESR	3.88 (1.36)	0.85	0.13**	0.33***	0.58***	0.42***	0.19***	0.08	0.18***	0.19***	0.05	0.36***	0.66***	0.47***	0.20***	0.10	0.13*	0.13*	0.06	0.39***	1				
20. INR	4.09 (1.36)	0.82	0.01	0.29***	0.39***	0.59***	0.39***	0.19***	0.16**	0.07	−0.08	0.29***	0.43***	0.63***	0.38***	0.25***	0.13*	0.08	−0.01	0.34***	0.48***	1			
21. IDR	4.66 (1.47)	0.90	−0.23***	0.07	0.21***	0.34***	0.59***	0.53***	−0.11*	−0.12*	−0.31***	0.18***	0.20***	0.36***	0.70***	0.59***	−0.15**	−0.09	−0.34***	0.21***	0.25***	0.52***	1		
22. IM	4.64 (1.51)	0.94	−0.27***	0.04	0.11*	0.19***	0.52***	0.72***	−0.25***	−0.18***	−0.36***	0.11*	0.07	0.24***	0.63***	0.75***	−0.25***	−0.13*	−0.45***	0.14**	0.17***	0.28***	0.71***	1	
23. UN	2.86 (0.93)	0.89	0.39***	0.12*	0.11*	0.10*	−0.16**	−0.35***	0.62***	0.49***	0.32***	0.07	0.13*	0.10	−0.21***	−0.38***	0.67***	0.58***	0.39***	0.08	0.09	0.15**	−0.21***	−0.37***	1
24. UR	2.61 (0.92)	0.90	0.29***	0.08	0.12*	0.08	−0.12**	−0.23***	0.47***	0.61***	0.28***	0.06	0.12*	0.05	–13*	−0.28***	0.47***	0.68***	0.34***	0.07	0.11	0.09	−0.14**	−0.24***	0.69***

*Note:* All statistics based on composite scores.

Abbreviations: ω, McDonald's omega; AM, amotivation; EMR, external material regulation; ESR, external social regulation; IDR, identified regulation; IM, intrinsic motivation; INR, introjected regulation; M, mean; SD, standard deviation; T, measurement point; UN, unnecessary tasks; UR, unreasonable tasks.

* *p* < 0.05.

** *p* < 0.01.

*** *p* < 0.001.

### 3.2 Measurement Models

The measurement models for MWMS had at least satisfactory fit except for the three‐factor model. For the first research question, the six‐factor model was chosen. Its configural, metric, and scalar invariance were supported in successive comparisons. Indices of the scalarly invariant six‐factor model indicated good fit (*χ* ^2^ = 1601.388, df = 1316, *p* < 0.001, CFI = 0.986, TLI = 0.983, SRMR = 0.028, RMSEA = 0.018; see full information on the models and invariance testing in Table 2).

**TABLE 2:** Longitudinal measurement models for MWMS.

Model	*χ* ^2^, df, *p*	CFI	TLI	SRMR	RMSEA (90% CI)	Scaling correction	Δ*χ* ^2^, Δdf, *p*	ΔCFI	ΔRMSEA
*Six‐factor model: amotivation, external material regulation, external social regulation, introjected regulation, identified regulation, intrinsic motivation*									
Configural invariance	1419.316, 1134, < 0.001	0.986	0.981	0.023	0.019 (0.016; 0.022)	1.1235	—	—	—
Metric invariance	1570.524, 1290, < 0.001	0.986	0.983	0.028	0.018 (0.014; 0.021)	1.1313	153.10, 156, 0.546	0.000	−0.001
Scalar invariance	1601.388, 1316, < 0.001	0.986	0.983	0.028	0.018 (0.014; 0.021)	1.1285	30.753, 26, 0.238	0.000	−0.000
*Five‐factor model, amotivation, external material regulation, external social regulation, introjected regulation, autonomous motivation (identified regulation and intrinsic motivation combined)*									
Configural invariance	2104.752, 1209, < 0.001	0.957	0.943	0.029	0.032 (0.030; 0.035)	1.1155	—	—	—
Metric invariance	2216.607, 1349, < 0.001	0.958	0.950	0.034	0.030 (0.028; 0.032)	1.1287	123.951, 140, 0.831	0.001	−0.002
Scalar invariance	2247.255, 1377, < 0.001	0.958	0.951	0.034	0.030 (0.028; 0.032)	1.1261	23.908, 28, 0.686	0.000	0.000
*Four‐factor model: amotivation, external regulation (external material regulation and external social regulation combined), introjected regulation, autonomous motivation (identified regulation and intrinsic motivation combined)*									
Configural invariance	2844.449, 1281, < 0.001	0.924	0.905	0.049	0.042 (0.040; 0.044)	1.1237	—	—	—
Metric invariance	2932.749, 1401, < 0.001	0.926	0.915	0.051	0.039 (0.037; 0.041)	1.1340	104.047, 120, 0.850	0.002	0.010
Scalar invariance	2970.378, 1431, < 0.001	0.925	0.917	0.052	0.039 (0.037; 0.041)	1.1309	33.934, 30, 0.283	−0.001	0.002
*Three‐factor model: amotivation, controlled motivation (external material regulation, external social regulation and introjected regulation combined), autonomous motivation (identified regulation and intrinsic motivation combined)*									
Configural invariance^a^	4221.431, 1350, < 0.001	0.861	0.835	0.060	0.055 (0.053; 0.057)	1.1380	—	—	—

*Note:* Δ indicates the change from the previous model.

^a^ Testing was discontinued after less than satisfactory fit.

Two latent illegitimate factors from BITS were added to the six‐factor model of MWMS and tested for configural, metric, and scalar invariance. Invariance was supported, and the final scalarly invariant measurement model for the first research question achieved good fit (*χ* ^2^ = 3848.197, df = 2813, *p* < 0.001, CFI = 0.966, TLI = 0.961, SRMR = 0.037, RMSEA = 0.023; see full information on the models and invariance testing in Table 3).

**TABLE 3:** Longitudinal measurement model of six regulatory style factors and two illegitimate task factors).

Model	*χ* ^2^, df, *p*	CFI	TLI	SRMR	RMSEA (90% CI)	Scaling correction	Δ*χ* ^2^, Δdf, *p*	ΔCFI	ΔRMSEA
Configural invariance	3633.641, 2607, < 0.001	0.966	0.958	0.035	0.024 (0.022; 0.025)	1.0860	—	—	—
Metric invariance	3793.443, 2775, < 0.001	0.967	0.961	0.037	0.023 (0.021; 0.025)	1.0923	165.910, 168, 0.531	0.001	0.003
Scalar invariance	3848.197, 2813, < 0.001	0.966	0.961	0.037	0.023 (0.021; 0.025)	1.0908	55.067, 38, 0.036	−0.001	0.000

*Note:* Δ indicates the change from previous model.

For the second research question, the five‐factor model of MWMS with factors for amotivation, external material regulation, external social regulation, introjected regulation, and autonomous motivation (identified regulation and intrinsic motivation combined) was chosen. The six‐factor model had somewhat better fit, but its factor correlations suggested potential multicollinearity problems (*r*'s between identified regulation and intrinsic motivation from 0.70 to 0.76 depending on measurement point). The scalarly invariant five‐factor model had a good fit (Table 2) and its factor correlations did not indicate as severe a risk for multicollinearity (strongest cross‐sectional correlations were between the two external regulatory styles, *r*'s from 0.50 to 0.60).

For cross‐lagged panel models in the second research question, a model with five regulatory style factors and one illegitimate task factor was chosen over a model with two related illegitimate task factors. The model with one illegitimate task factor had good fit and not as severe a risk for multicollinearity as the model with two illegitimate task factors (in the model with two illegitimate task factors, cross‐sectional *r*'s between the factors were from 0.68 to 0.74). See Table 4 for complete information on fit and measurement invariance statistics.

**TABLE 4:** Longitudinal measurement model of five regulatory style factors and one or two illegitimate task factors.

Model	*χ* ^2^, df, *p*	CFI	TLI	SRMR	RMSEA (90% CI)	Scaling correction	Δ*χ* ^2^, Δdf, *p*	ΔCFI	ΔRMSEA
*Five regulatory style factors and one illegitimate task factor*									
Configural invariance	2464.203, 1506, < 0.001	0.958	0.945	0.030	0.030 (0.028; 0.032)	1.0995	—	—	—
Metric invariance	2580.642, 1648, < 0.001	0.959	0.952	0.034	0.028 (0.026; 0.030)	1.1118	128.611, 142, 0.783	0.001	−0.002
Scalar invariance	2616.776, 1678, < 0.001	0.959	0.952	0.035	0.028 (0.026; 0.030)	1.1097	34.876, 30, 0.247	0.000	0.000
*Five regulatory style factors and two illegitimate task factors*									
Configural invariance	4359.414, 2700, < 0.001	0.945	0.935	0.037	0.030 (0.028; 0.031)	1.0834	—	—	—
Metric invariance	4479.868, 2852, < 0.001	0.947	0.939	0.040	0.028 (0.027; 0.030)	1.0915	135.008, 152, 0.835	0.002	−0.002
Scalar invariance	4533.041, 2892, < 0.001	0.946	0.940	0.040	0.028 (0.027; 0.030)	1.0903	52.351, 40, 0.091	−0.001	0.000

*Note:* Δ indicates the change from previous model.

### 3.3 Associations Between Illegitimate Tasks and Regulatory Styles

According to Hypothesis 1, illegitimate tasks are associated with regulatory styles in a predictable manner. Fixing covariances between illegitimate task factor regulatory style factors equal across measurement points did not decrease fit (Δ*χ* ^2^ = 31.103, Δdf = 24 *p* = 0.151). Similarly, fixing the covariances between each respective regulatory style and both illegitimate task factors equal did not decrease fit (Δ*χ* ^2^ = 25.638, Δdf = 6, *p* < 0.001, ΔCFI = −0.001, ΔRMSEA = 0.000). This suggests that the associations are not affected by the measurement occasion and that there are no differential associations between unnecessary and unreasonable tasks and regulatory styles.

The factor correlations from the retained model with their 95% CIs are presented by measurement point in Table 5. Hypothesis 1a was supported because the correlation between illegitimate tasks and low intrinsic motivation was close to expectations (*r*'s from −0.25 to −0.28 versus the hypothesized *r* ≈ −0.30). Hypothesis 1b was also supported because the association between identified regulation and illegitimate tasks was close to the hypothesis, although the association may not be robustly as large as was expected (*r*'s from −0.14 to −0.16 versus the hypothesized *r* ≈ −0.20).

**TABLE 5:** Correlations (95% confidence intervals) between regulatory styles and illegitimate task.

	Time 1		Time 2		Time 3	
	Unnecessary tasks	Unreasonable tasks	Unnecessary tasks	Unreasonable tasks	Unnecessary tasks	Unreasonable tasks
Amotivation	0.46 (0.40; 0.52)	0.46 (0.40; 0.52)	0.45 (0.39; 0.51)	0.44 (0.38; 0.50)	0.48 (0.41; 0.55)	0.43 (0.37; 0.49)
External material regulation	0.17 (0.09; 0.25)	0.17 (0.09; 0.25)	0.17 (0.09; 0.25)	0.17 (0.09; 0.24)	0.17 (0.09; 0.25)	0.16 (0.08; 0.23)
External social regulation	0.17 (0.10; 0.24)	0.17 (0.10; 0.24)	0.17 (0.10; 0.25)	0.17 (0.10; 0.24)	0.17 (0.10; 0.24)	0.15 (0.09; 0.21)
Introjected regulation	0.20 (0.12; 0.27)	0.20 (0.12; 0.27)	0.20 (0.12; 0.26)	0.20 (0.12; 0.26)	0.20 (0.13; 0.28)	0.18 (0.11; 0.25)
Identified regulation	−0.16 (−0.23; −0.08)	−0.16 (−0.23; −0.08)	−0.16 (−0.24; −0.08)	−0.16 (−0.23; −0.08)	−0.16 (−0.24; −0.08)	−0.14 (−0.21; −0.07)
Intrinsic motivation	−0.28 (−0.35; −0.21)	−0.28 (−0.35; −0.21)	−0.28 (−0.35; −0.20)	−0.27 (−0.34; −0.20)	−0.28 (−0.36; −0.21)	−0.25 (−0.32; −0.19)

*Note:* Correlation coefficient from the scalarly invariant model, correlations between illegitimate tasks and regulatory styles fixed equal cross‐sectionally and across time points (*χ* ^2^ = 3904.77, df = 2843, CFI = 0.965, TLI = 0.960, SRMR = 0.042, RMSEA = 0.023). All correlations statistically significant at *p* < 0.001.

Hypothesis 1c was not supported. Although the direction of the association between illegitimate tasks and introjected regulation was as expected, its magnitude was consistently larger than was hypothesized (*r*'s from 0.18 to 0.20 versus hypothesized *r* ≈ 0.10). Hypothesis 1d was supported because the associations between illegitimate tasks and external regulatory styles were as expected (*r*'s from 0.15 to 17 versus the hypothesized *r* ≈ 0.20). Finally, Hypothesis 1e could be considered supported, although the association between illegitimate tasks and amotivation was somewhat stronger than hypothesized (*r*'s from 0.44 to 0.48 versus the hypothesized *r* ≈ 0.30).

### 3.4 Temporal Relations Between Illegitimate Tasks and Regulatory Styles

According to Hypothesis 2, based on the SDT model in the workplace (Deci et al. 2017), illegitimate tasks act as a stressor and as an antecedent to regulatory styles (an illegitimate tasks to behavioral regulatory styles model). More specifically, according to Hypothesis 2, illegitimate tasks predict low autonomous motivation and high amotivation. An autoregressive model had satisfactory fit (*χ* ^2^ = 3035.633, df = 1871, *p* < 0.001, CFI = 0.949, TLI = 0.944, SRMR = 0.057, RMSEA = 0.030) and constraining the predictive paths to be equal did not decrease fit (Δ*χ* ^2^ = 11.633, Δdf = 6, *p* = 0.070). Specifying predictive paths from illegitimate tasks to behavioral regulations increased fit (Δ*χ* ^2^ = 18.431, Δdf = 10, *p* = 0.048) and setting them equal across time did not decrease fit (Δ*χ* ^2^ = 7.232, Δdf = 5, *p* = 0.204). Therefore, the hypothesized illegitimate tasks to needs model was initially supported. See Table 6 for full information on fit statistics and model comparisons.

**TABLE 6:** Comparison of predictive models.

Model	*χ* ^2^, df, *p*	CFI	TLI	SRMR	RMSEA (90% CI)	Scaling correction	Δ*χ* ^2^, Δdf, *p*	ΔCFI	ΔRMSEA
Autoregressive model	3035.633, 1865, < 0.001	0.949	0.944	0.057	0.030 (0.028; 0.032)	1.1055	—	—	—
Autoregressive model with equilibrium	3048.562, 1871, < 0.001	0.949	0.944	0.057	0.030 (0.028; 0.032)	1.1060	11.663, 6, 0.070	0.000	0.000
Illegitimate tasks to regulatory styles model	3029.842, 1861, < 0.001	0.950	0.944	0.053	0.030 (0.028; 0.032).	1.1052	18.431, 10, 0.048	0.001	0.000
Illegitimate tasks to regulatory styles model with equilibrium	3036.829, 1866, < 0.001	0.949	0.944	0.054	0.030 (0.028; 0.032).	1.1060	7.232, 5, 0.204	−0.001	0.000
^a^Regulatory styles to illegitimate tasks model	3025.834, 1861, < 0.001	0.950	0.944	0.055	0.030 (0.028; 0.032)	1.1060	27.728, 10, 0.012	0.001	0.000
Regulatory styles to illegitimate tasks model with equilibrium	3032.128, 1866, < 0.001	0.950	0.944	0.056	0.030 (0.028; 0.032)	1.1063	6.462, 5, 0.264	0.000	0.000
^a^Reciprocal model	3014.743, 1856, < 0.001	0.950	0.944	0.052	0.030 (0.028; 0.032)	1.1054	33.224, 15, 0.004	0.001	0.000
Reciprocal model with equilibrium	3021.290, 1861, < 0.001	0.950	0.944	0.052	0.030 (0.028; 0.032)	1.1062	6.880, 5, 0.230	0.000	0.000

*Note:* Δ indicates the change from the previous model. All models are compared with the previous model unless noted otherwise.

^a^ Compared with the autoregressive model with equilibrium.

An alternative model of behavioral regulations to illegitimate tasks was also tested. Predictive paths from regulations to illegitimate tasks increased fit when comparing to the autoregressive equilibrium model (Δ*χ* ^2^ = 27.728, Δdf = 10, *p* = 0.012) and setting them equal across time did not decrease fit (Δ*χ* ^2^ = 6.462, Δdf = 5, *p* = 0.264). Thus, this model was also initially supported. See Table 6 for full information on fit statistics and model comparisons.

Finally, a reciprocal model with predictive paths from illegitimate tasks to behavioral regulations and from regulations to illegitimate tasks was tested. Predictive paths increased fit compared to the autoregressive equilibrium model (Δ*χ* ^2^ = 33.224, Δdf = 15, *p* = 0.004), and setting them equal across time did not decrease fit (Δχ^2^ = 6.880, Δdf = 5, *p* = 0.230). The reciprocal model with equilibrium had satisfactory fit (*χ* ^2^ = 3021.290, df = 1861, *p* < 0.001, CFI = 0.950, TLI = 0.944, SRMR = 0.052, RMSEA = 0.030) and was chosen as the final model. Thus, although Hypothesis 2 was partially supported, the reciprocal model with equilibrium demonstrated the best fit. See Table 6 for full information on fit statistics and model comparisons.

The model was further examined regarding statistically significant predictive paths. Among all factors, temporal stability was high (autoregressive *β*'s from 0.63 to 0.79). Regarding cross‐lagged associations from illegitimate tasks to behavioral regulations, perceived illegitimate tasks predicted increasing amotivation (*β* = 0.08 at T2 and *β* = 0.09 at T3, both *p* < 0.05), and decreasing autonomous motivation (*β* = −0.05 at T2 and T3, both *p* < 0.05). Cross‐lagged associations from behavioral regulations to illegitimate tasks suggested that introjected regulation increased perceiving illegitimate tasks (*β* = 0.07 at T1 and *β* = 0.06 at T2, both *p* < 0.05), and that autonomous motivation decreased perceiving illegitimate tasks (*β* = −0.11 at T1 and *β* = −0.10 at T2, both *p* < 0.001). Therefore, in addition to illegitimate tasks predicting high amotivation and low autonomous motivation, some regulatory styles predicted perceiving illegitimate tasks. See full information on the final model's path coefficients in Table 7.

**TABLE 7:** Parameter estimates from the final predictive model (reciprocal model with equilibrium).

Predictor → Outcome	B (SE)	T1 → T2	T2 → T3
		*β* (*p*)	*β* (*p*)
*Autoregressive paths*			
Illegitimate tasks → Illegitimate tasks	0.79 (0.05)	0.77 (< 0.001)	0.76 (< 0.001)
Amotivation → Amotivation	0.64 (0.05)	0.65 (< 0.001)	0.67 (< 0.001)
External material regulation → External material regulation	0.74 (0.04)	0.75 (< 0.001)	0.79 (< 0.001)
External social regulation → External social regulation	0.68 (0.04)	0.66 (< 0.001)	0.72 (< 0.001)
Introjected regulation → Introjected regulation	0.65 (0.04)	0.70 (< 0.001)	0.63 (< 0.001)
Autonomous motivation → Autonomous motivation	0.75 (0.02)	0.76 (< 0.001)	0.76 (< 0.001)
*Cross‐lagged paths from illegitimate tasks to regulatory styles*			
Illegitimate tasks → Amotivation	0.08 (0.04)	0.08 (0.013)	0.09 (0.015)
Illegitimate tasks → External material regulation	0.02 (0.03)	0.02 (0.232)	0.02 (0.233)
Illegitimate tasks → External social regulation	0.04 (0.03)	0.04 (0.091)	0.04 (0.089)
Illegitimate tasks → Introjected regulation	−0.02 (0.03)	−0.03 (0.760)	−0.03 (0.760)
Illegitimate tasks → Autonomous motivation	−0.05 (0.02)	−0.05 (0.022)	−0.05 (0.021)
*Cross‐lagged paths from regulatory styles to illegitimate tasks*			
Amotivation → Illegitimate tasks	−0.01 (0.04)	−0.01 (0.579)	−0.01 (0.569)
External material regulation → Illegitimate tasks	−0.04 (0.04)	−0.04 (0.856)	−0.04 (0.857)
External social regulation → Illegitimate tasks	−0.00 (0.04)	−0.00 (0.546)	−0.00 (0.546)
Introjected regulation → Illegitimate tasks	0.07 (0.04)	0.07 (0.035)	0.06 (0.035)
Autonomous motivation → Illegitimate tasks	−0.12 (0.04)	−0.11 (< 0.001)	−0.10 (< 0.001)

*Note:* Model fit: *χ* ^2^ = 3021.290, df = 1861, *p* < 0.001, CFI = 0.950, TLI = 0.944, SRMR = 0.052, RMSEA = 0.030 (90% CI = 0.028; 0.032). Regression coefficients of age and gender are not shown.

Abbreviations: *β*, standardized regression coefficient; B, unstandardized regression coefficient that are fixed invariant between measurement points; *p*, statistical significance of the standardized regression coefficient based on 1‐tailed testing; SE, standard error the unstandardized regression coefficient; T1, first measurement point; T2, second measurement point; T3, third measurement point.

## 4 Discussion

### 4.1 Main Findings and Their Theoretical Implications

There are two main findings. According to the first, associations between illegitimate tasks and behavioral regulatory styles do not differ according to facets of illegitimate tasks, are mostly theoretically comprehensible, and are consistent between the measurement points. According to the second main finding, temporal relationships of illegitimate tasks and behavioral regulations follow a model of reciprocal associations.

Regarding the first main finding and the least self‐determined regulatory style, the association between illegitimate tasks and amotivation was consistently positive, with a relatively large effect size. The association was expected because illegitimate tasks, by definition, are involuntary duties assigned by others (Semmer et al. 2019; Muntz and Dormann 2020), have a low utility value to the core tasks (Ryan and Deci 2017), and signal disrespect. It seems that working in an environment with a lot of perceived illegitimate tasks is close to the experience of acting with no personal intention or non‐volitionally.

Moving onto more self‐determined regulatory styles, the associations between illegitimate tasks and external regulation (both material and social) and introjected regulation were positive. It was hypothesized that the association between introjected regulation and illegitimate tasks would be smaller than that between illegitimate tasks and external regulation, but there were no appreciable differences in the magnitude of associations. It seems that the pronounced experience of contingencies matters more than whether the contingencies are external, as in external regulatory styles, or partially internalized, as in introjected regulatory style. Speculatively, the association between external regulation and illegitimate tasks could also depend on the significance of the latter to external rewards and punishments. Provided that carrying out illegitimate tasks is a monitored necessity for such external incentives as job security, bonus payment, or social approval, the association could be even of large magnitude. However, if illegitimate tasks are an aspect of one's job that is not consistently overseen, their relationship with external regulation could be considerably smaller. Contextual features moderating the association between illegitimate tasks and external regulatory styles could be examined in further studies.

Finally, regarding the first main finding and the most self‐determined regulatory styles, the associations between illegitimate tasks and both identified regulation and intrinsic motivation were negative. I hypothesized that the association between intrinsic motivation and illegitimate tasks would be stronger than the association between illegitimate tasks and identified regulation. Overall, the correlations were consistent with this suggestion. The strength of the associations with intrinsic motivation corroborates some of the previous observations (Fila et al. 2023; Omansky et al. 2016) and suggests that some results of Muntz and Dormann (2020) may be due to differences in measurement. The results of the present study imply that, with more illegitimate tasks, it is more difficult to endorse or value one's actions within one's job as part of one's coherent self (low identified regulation), and it is improbable to put so much effort into it for enjoyment and interest (low intrinsic motivation). Similar to external regulations, it can be speculated if there are factors moderating associations between illegitimate tasks and identified regulation. For example, defensive processes such as the compartmentalization of identifications (particular identifications remaining actively and defensively unintegrated; Ryan and Deci 2017) or flexible role orientation (defining one's role broadly and in proactive terms; Parker 2007; Ma and Peng 2019) could facilitate maintaining or developing strong identifications with the job despite some tasks being perceived as illegitimate. With defensive compartmentalization, an employee could perhaps metaphorically put illegitimate tasks in a mental compartment separate from other tasks and still be able to have stronger identification with the job as a whole. An employee with a flexible role orientation can potentially interpret their work role so broadly that they can see the individual‐level illegitimate tasks as only one part of their job that may have significance at the level of team and organization. However, these are all speculations for now, because no mediating or moderating factors were assessed.

According to the second main finding, perceived illegitimate tasks and behavioral regulations have a reciprocal longitudinal relationship. Examination of this relationship is appropriate to begin with the extreme ends of the self‐determination continuum. Perceived illegitimate tasks predicted higher amotivation and lower autonomous motivation (indicated by items of both identified regulation and intrinsic motivation) over time, and autonomous motivation predicted lower illegitimate tasks over time. If interpreted causally, this dynamic implies that perceptions of task illegitimacy erode motivational quality by making it less self‐determined, but autonomous motivation is a resource that reduces experienced illegitimacy. Frameworks such as SOS theory (Semmer et al. 2019) would explain the potential influence of illegitimate tasks on motivation by considering illegitimate tasks as stressors that affect motivation and well‐being via, for example, self‐evaluative and energy‐depleting processes. From the perspective of SDT model in the workplace (Deci et al. 2017), satisfaction of basic psychological needs would be a key explanatory factor in all such processes.

However, the potential influence of autonomous motivation on decreasing illegitimate tasks requires additional considerations. There are at least two possible explanations for the association. The first one is a perceptual change mechanism De Lange et al. (2005) call “a rosy perception mechanism” (see also Muntz and Dormann 2020). The mechanism suggests processes through which work demands are experienced as more meaningful, conducive to self‐expression, and easier to assimilate with one's values. High autonomous motivation may facilitate experiencing the assigned tasks as more meaningful and easier to integrate with other tasks and the job as a whole. The other possible mechanism considers the antecedents of a more self‐determined motivational experience. Several job resources, such as skill variety, job autonomy, and social support, are associated with basic psychological need satisfaction (Van den Broeck et al. 2016). Similarly, job crafting has been suggested to predict autonomous motivation (Li et al. 2021). It is possible that job resources or active job crafting allow an employee to affect the content or methods by which their job can be carried out, including the decrease of illegitimate tasks. With this mechanism, regulatory styles would not have any causal role. Both the decrease in perceived illegitimate tasks and more self‐determined work motivation would be consequences of acquiring or having and utilizing appropriate job resources. However, testing the potential mechanisms requires variables not examined in the present study.

In addition to associations discussed above, introjected regulation predicted an increase in illegitimate tasks over time. Introjected regulatory style corresponds to a somewhat conflicted and tense motivational experience (Ryan and Deci 2017). The drivers of behavior, and affective and evaluative contingencies are partially internalized. It can be speculated that this partial and conflicted internalization activates attempts to further internalize experiences in ways that the more external regulatory styles do not. For example, perhaps a strong external material regulatory style does not predict perceiving more illegitimate tasks over time because an employee's attitude to tasks is very instrumental, and the perceived illegitimacy of assigned tasks does not appreciably vary as long as the employee gets paid. With a strong introjected regulatory style and accompanied contingent self‐worth, it may be more difficult to ignore stress when potentially illegitimate tasks are assigned. It is noteworthy that Muntz and Dormann (2020) observed that intrinsic motivation predicted more perceived illegitimate tasks assigned by colleagues over two weeks. I argued that the contents of the measure they used correspond better to the definition of introjected regulation in SDT. Should my estimate be correct, the introjected regulatory style has been found to predict an increasing perception of illegitimate tasks in both the short term in Muntz and Dormann's study, and in the longer term in the present study. Nevertheless, replications are needed.

### 4.2 Evaluation of the Study

The present study has some limitations. First, constructing exact hypotheses on the magnitude of associations may not be suitable or sensible in psychological research. Previous observations (Fila et al. 2023; Omansky et al. 2016) facilitated setting expectations for intrinsic motivation and an upper limit for the other correlations. However, most of the hypothesized strengths of the associations in the present study were based on the degree of self‐determination of the regulatory styles. Nevertheless, the theoretical background of the study was strong and facilitates further research with an explicated outline of the expected associations.

Second, there is no certainty on the optimal time lag between the measurement points. Muntz and Dormann (2020) used a one‐week lag, whereas in the present study, the lag was four months. Different measurement intervals could produce different results, and it is advisable to test different time lags in further studies.

Third, attrition was higher especially among the respondents with high amotivation at the beginning of the study. This may have decreased the range of scores for some variables. However, the associations between amotivation and illegitimate tasks at the different measurement points were very similar. Additionally, attrition among the participants with fixed‐term contracts could hinder generalizability.

Fourth, several factors were highly correlated both cross‐sectionally and longitudinally, which could lead to problems with multicollinearity in the regression analyses. The correlations were highest between the unreasonable tasks and unnecessary tasks and between identified regulation and intrinsic motivation. In many previous studies, illegitimate tasks were operationalized as a single factor comprising the facet scales as indicators (e.g., Omansky et al. 2016; Semmer et al. 2010), but in several other studies, both factors were used (e.g., Fila and Eatough 2020; Mäkikangas et al. 2023; Pindek et al. 2019). I decided to use different measurement models in different analyses. When examining correlative associations (the first research question), six behavioral regulatory styles and two factors of illegitimate tasks were used. When temporally predictive models were tested and multicollinearity might have been detrimental to the reliability of the results (the second research question), a model with five regulatory style factors and one illegitimate tasks factor was used. Although this resolution may seem indecisive, it facilitates providing analyses that are both maximally informative and statistically less biased. For example, although identified regulation and intrinsic motivation are empirically very strongly associated, identified regulation has a component of regulatory forces external to the activity itself, which intrinsic motivation does not have. Estimating the factors as separate but related made it possible to examine the potentially differential associations with illegitimate tasks in correlational analyses. However, the same approach could have biased estimates in regression analyses due to multicollinearity, and therefore indicators of identified regulation and intrinsic motivation formed a factor of autonomous motivation. Additionally, it would have been theoretically sound to estimate a single factor of extrinsic regulation, indicated by items of both extrinsic material regulation and extrinsic social regulation. Admittedly, the decision to model two extrinsic regulatory style factors was more driven by the better relative fit of the five‐factor model compared to the four‐factor model. According to the present study, estimating the same measurement models for different research questions, and as few distinct regulatory style factors as are theoretically and empirically justified, is recommended for further studies. It should also be noted that because MWMS omits the integrated regulatory style, every qualitatively distinct aspect of the self‐determination continuum is not assessed in the present study. Despite this, the continuum was examined from amotivation to intrinsic motivation through several external regulatory styles, providing a fuller picture of the subject matter compared to previous studies.

Fifth, many potential mechanisms for the associations were speculated but not tested. Job resources such as job autonomy, social support, and leadership styles as determinants of both satisfaction of basic psychological needs and behavioral regulatory styles should be tested in further studies. Similarly, some job demands would have been important to control for. For example, many employees who have a lot of tasks they think are illegitimate probably have a high workload in general.

The present study also has strengths regarding the sample, design, method, and theoretical basis. A large and heterogeneous sample was collected from the working population. A longitudinal design with a relatively good response rate and attempts to assess careful responding facilitates generalizability, although replications are welcome. The ESEM method used to examine the motivational structure with underlying dimensions was theoretically sound, and its use alleviates some concerns about multicollinearity (Howard 2023; Howard et al. 2018; Marsh et al. 2014). The study has a firm basis (SDT, previous empirical observations and partly the SOS theory), making it possible to test some reasonable hypotheses and generate new ones.

### 4.3 Practical Implications and Suggestions for Further Research

The cross‐sectional associations between regulatory styles and illegitimate tasks indicate that strong intrinsic motivation is a relatively rare experience when several tasks are perceived as illegitimate (the opposite is true with amotivation). This finding should encourage managers and other stakeholders responsible for task assignments and overseeing employee performance to determine how employees interpret task illegitimacy. Regardless of the potential temporal and causal dynamics, which are difficult to demonstrate, listening to one's employees and trying to understand their reasons for experiencing amotivation or perceiving some tasks as illegitimate is of utmost importance.

The findings on potential temporal relationships imply some courses of action. First, perceived illegitimate tasks predicted an increase in amotivation and a decrease in autonomous motivation, and autonomous motivation predicted a decrease in perceived illegitimate tasks. If autonomous work motivation facilitates seeing assigned tasks as less illegitimate, several job resources that support more self‐determined regulatory styles can indirectly, through work motivation or potentially even directly, facilitate experiencing fewer illegitimate tasks. The most potential determinants of autonomous motivation are satisfaction of the basic psychological needs and leader autonomy support (Slemp et al. 2018).

Second, although the hypothetically detrimental effects of illegitimate tasks could be alleviated with defensive compartmentalizations or a flexible role orientation, these are barely compensatory means. Efforts should be put into reducing the number of tasks that are justifiably perceived as illegitimate or into helping the employee make sense of the seemingly illegitimate tasks.

The findings allow several suggestions for further research to be made. Of course, both the cross‐sectional and longitudinal observations should be replicated. Furthermore, the several speculations could be presented but not studied with the current data. For example, it was suggested that the strength of the association between external regulation and illegitimate tasks could depend on the monitoring of the accomplishment of illegitimate tasks and that the associations between illegitimate tasks and identified regulation could be affected by compartmentalizations of identity or other defensive processes.

The temporal relationship between illegitimate tasks and regulatory styles must be tested in future studies, and additional antecedents of both must be examined. Potential candidates for the causes and consequences of the focal constructs of the present study are basic psychological need satisfaction and several organization‐related factors, such as leadership style and organizational politics.

## 5 Conclusion

Based on the results of the present study, illegitimate tasks are largely predictably associated with the quality of work motivation. High amotivation and low intrinsic motivation showed the strongest and most consistent associations with illegitimate tasks, with the associations with the other regulatory styles being more moderate. Additionally, they have consistent and reciprocal temporal associations. Most importantly, perceived illegitimate tasks predict an increase in amotivation and a decrease in autonomous motivation, and autonomous motivation predicts a decrease in perceived illegitimate tasks over time. Reducing illegitimate tasks may be an important factor in maintaining more self‐determined work motivation, and maintaining autonomous motivation may be one way to avoid the accumulation of illegitimate tasks.

## Author Contributions

Petri Karkkola: conceptualisation, data curation, formal analysis, funding acquisition, methodology, original draft preparation, review and editing.

## Conflicts of Interest

The author declares no conflicts of interest.

## Data Availability

Data are available upon reasonable request. Data will be archived according to the data management plan.

## References

[sjop70025-bib-0001] Apostel, E. , C. J. Syrek , and C. H. Antoni . 2018. “Turnover Intention as a Response to Illegitimate Tasks: The Moderating Role of Appreciative Leadership.” International Journal of Stress Management 25, no. 3: 234–249.

[sjop70025-bib-0002] Bureau, J. S. , F. Guay , A. Plamondon , C. F. Ratelle , J. L. Howard , and W. Gilbert . 2023. “Empirical Testing of an Alternative Modeling of the Self‐Determination Continuum.” Motivation and Emotion 47, no. 1: 46–60. 10.1007/s11031-022-09976-9.

[sjop70025-bib-0003] Chen, F. F. 2007. “Sensitivity of Goodness of Fit Indexes to Lack of Measurement Invariance.” Structural Equation Modeling: A Multidisciplinary Journal 14, no. 3: 464–504. 10.1080/10705510701301834.

[sjop70025-bib-0004] Cregård, A. , and L. Corin . 2019. “Public Sector Managers: The Decision to Leave or Remain in a Job.” Human Resource Development International 22, no. 2: 158–176. 10.1080/13678868.2018.1563749.

[sjop70025-bib-0005] De Lange, A. H. , T. W. Taris , M. A. Kompier , I. L. Houtman , and P. M. Bongers . 2005. “Different Mechanisms to Explain the Reversed Effects of Mental Health on Work Characteristics.” Scandinavian Journal of Work, Environment & Health 31, no. 1: 3–14. 10.5271/sjweh.843. 15751614

[sjop70025-bib-0006] Deci, E. L. , A. H. Olafsen , and R. M. Ryan . 2017. “Self‐Determination Theory in Work Organizations: The State of a Science.” Annual Review of Organizational Psychology and Organizational Behavior 4, no. 1: 19–43. 10.1146/annurev-orgpsych-032516-113108.

[sjop70025-bib-0007] Ding, H. , and B. Kuvaas . 2023. “Illegitimate Tasks: A Systematic Literature Review and Agenda for Future Research.” Work & Stress 37, no. 3: 397–420. 10.1080/02678373.2022.2148308.

[sjop70025-bib-0008] Dunn, A. M. , E. D. Heggestad , L. R. Shanock , and N. Theilgard . 2018. “Intra‐Individual Response Variability as an Indicator of Insufficient Effort Responding: Comparison to Other Indicators and Relationships With Individual Differences.” Journal of Business and Psychology 33, no. 1: 105–121. 10.1007/s10869-016-9479-0.

[sjop70025-bib-0009] Fila, M. J. , and E. Eatough . 2020. “Extending the Boundaries of Illegitimate Tasks: The Role of Resources.” Psychological Reports 123, no. 5: 1635–1662. 10.1177/0033294119874292. 31510875

[sjop70025-bib-0010] Fila, M. J. , N. K. Semmer , and M. Kern . 2023. “When Being Intrinsically Motivated Makes You Vulnerable: Illegitimate Tasks and Their Associations With Strain, Work Satisfaction, and Turnover Intention.” Occupational Health Science 7, no. 2: 189–217. 10.1007/s41542-022-00140-w.

[sjop70025-bib-0011] Gagné, M. , J. Forest , M. Vansteenkiste , et al. 2015. “The Multidimensional Work Motivation Scale: Validation Evidence in Seven Languages and Nine Countries.” European Journal of Work and Organizational Psychology 24, no. 2: 178–196. 10.1080/1359432X.2013.877892.

[sjop70025-bib-0012] Garn, A. C. , A. J. S. Morin , and C. Lonsdale . 2019. “Basic Psychological Need Satisfaction Toward Learning: A Longitudinal Test of Mediation Using Bifactor Exploratory Structural Equation Modeling.” Journal of Educational Psychology 111, no. 2: 354–372. 10.1037/edu0000283.

[sjop70025-bib-0013] Howard, J. L. 2023. “Psychometric Approaches in Self‐Determination Theory: Meaning and Measurement.” In The Oxford Handbook of Self‐Determination Theory, edited by R. M. Ryan , 1st ed., 438–454. Oxford University Press. 10.1093/oxfordhb/9780197600047.013.15.

[sjop70025-bib-0014] Howard, J. L. , M. Gagné , and J. S. Bureau . 2017. “Testing a Continuum Structure of Self‐Determined Motivation: A Meta‐Analysis.” Psychological Bulletin 143, no. 12: 1346–1377. 10.1037/bul0000125. 29048175

[sjop70025-bib-0015] Howard, J. L. , M. Gagné , A. J. S. Morin , and J. Forest . 2018. “Using Bifactor Exploratory Structural Equation Modeling to Test for a Continuum Structure of Motivation.” Journal of Management 44, no. 7: 2638–2664. 10.1177/0149206316645653.

[sjop70025-bib-0016] Howard, J. L. , M. Gagné , A. Van den Broeck , et al. 2020. “A Review and Empirical Comparison of Motivation Scoring Methods: An Application to Self‐Determination Theory.” Motivation and Emotion 44, no. 4: 534–548. 10.1007/s11031-020-09831-9.

[sjop70025-bib-0017] Kilponen, K. , M. Huhtala , U. Kinnunen , S. Mauno , and T. Feldt . 2021. “Illegitimate Tasks in Health Care: Illegitimate Task Types and Associations With Occupational Well‐Being.” Journal of Clinical Nursing 30, no. 13–14: 2093–2106. 10.1111/jocn.15767. 33829574

[sjop70025-bib-0018] Kronenwett, M. , and T. Rigotti . 2019. “When Do You Face a Challenge? How Unnecessary Tasks Block the Challenging Potential of Time Pressure and Emotional Demands.” Journal of Occupational Health Psychology 24, no. 5: 512–526. 10.1037/ocp0000149. 30945921

[sjop70025-bib-0019] Landers, R. N. 2020. “*Calculating LongString in Excel to Detect Careless Responders* [Preprint].” 10.22541/au.160590023.35753324/v1.

[sjop70025-bib-0020] Li, Y. , X. Li , and Y. Liu . 2021. “How Does High‐Performance Work System Prompt Job Crafting Through Autonomous Motivation: The Moderating Role of Initiative Climate.” International Journal of Environmental Research and Public Health 18, no. 2: 384. 10.3390/ijerph18020384. 33419076 PMC7825400

[sjop70025-bib-0021] Little, T. D. 2013. Longitudinal Structural Equation Modeling. Guilford Press.

[sjop70025-bib-0022] Ma, J. , and Y. Peng . 2019. “The Performance Costs of Illegitimate Tasks: The Role of Job Identity and Flexible Role Orientation.” Journal of Vocational Behavior 110: 144–154. 10.1016/j.jvb.2018.11.012.

[sjop70025-bib-0023] Mäkikangas, A. , J. Minkkinen , J. Muotka , and S. Mauno . 2023. “Illegitimate Tasks, Job Crafting and Their Longitudinal Relationships With Meaning of Work.” International Journal of Human Resource Management 34, no. 7: 1330–1358. 10.1080/09585192.2021.1987956.

[sjop70025-bib-0024] Marsh, H. W. , A. J. S. Morin , P. D. Parker , and G. Kaur . 2014. “Exploratory Structural Equation Modeling: An Integration of the Best Features of Exploratory and Confirmatory Factor Analysis.” Annual Review of Clinical Psychology 10, no. 1: 85–110. 10.1146/annurev-clinpsy-032813-153700. 24313568

[sjop70025-bib-0025] Mauno, S. , J. Minkkinen , and A. Shimazu . 2022. “Do Unnecessary Tasks Impair Performance Because They Harm Living a Calling? Testing a Mediation in a Three‐Wave Study.” Journal of Career Assessment 30, no. 1: 94–109. 10.1177/10690727211018977.

[sjop70025-bib-0026] Meade, A. W. , and S. B. Craig . 2012. “Identifying Careless Responses in Survey Data.” Psychological Methods 17, no. 3: 437–455. 10.1037/a0028085. 22506584

[sjop70025-bib-0027] Morin, A. J. S. , H. W. Marsh , and B. Nagengast . 2013. “Exploratory Structural Equation Modeling.” In Structural Equation Modeling: A Second Course, edited by H. W. Marsh , 2nd ed., 395–436. IAP Information Age Publishing.

[sjop70025-bib-0028] Muntz, J. , and C. Dormann . 2020. “Moderating Effects of Appreciation on Relationships Between Illegitimate Tasks and Intrinsic Motivation: A Two‐Wave Shortitudinal Study.” European Journal of Work and Organizational Psychology 29, no. 3: 391–404. 10.1080/1359432X.2019.1706489.

[sjop70025-bib-0029] Omansky, R. , E. M. Eatough , and M. J. Fila . 2016. “Illegitimate Tasks as an Impediment to Job Satisfaction and Intrinsic Motivation: Moderated Mediation Effects of Gender and Effort‐Reward Imbalance.” Frontiers in Psychology 7: 1818. 10.3389/fpsyg.2016.01818. 27917145 PMC5116474

[sjop70025-bib-0030] Parker, S. K. 2007. “That Is My Job’: How Employees' Role Orientation Affects Their Job Performance.” Human Relations 60, no. 3: 403–434. 10.1177/0018726707076684.

[sjop70025-bib-0031] Pelletier, L. G. , and M. Rocchi . 2023. “Organismic Integration Theory: A Theory of Regulatory Styles, Internalization, Integration, and Human Functioning in Society.” In The Oxford Handbook of Self‐Determination Theory, edited by R. M. Ryan , 1st ed., 53–C3P192. Oxford University Press. 10.1093/oxfordhb/9780197600047.013.4.

[sjop70025-bib-0032] Pindek, S. , E. Demircioğlu , D. J. Howard , E. M. Eatough , and P. E. Spector . 2019. “Illegitimate Tasks Are Not Created Equal: Examining the Effects of Attributions on Unreasonable and Unnecessary Tasks.” Work & Stress 33, no. 3: 231–246. 10.1080/02678373.2018.1496160.

[sjop70025-bib-0033] Ryan, R. M. , and E. L. Deci . 2002. “Overview of Self‐Determination Theory: An Organismic Dialectical Perspective.” In Handbook of Self‐Determination Research, edited by E. L. Deci and R. M. Ryan , 3–33. University of Rochester Press.

[sjop70025-bib-0034] Ryan, R. M. , and E. L. Deci . 2017. Self‐Determination Theory: Basic Psychological Needs in Motivation, Development, and Wellness. Guilford Press.

[sjop70025-bib-0035] Ryan, R. M. , and M. Vansteenkiste . 2023. “Self‐Determination Theory: Metatheory, Methods, and Meaning.” In The Oxford Handbook of Self‐Determination Theory, edited by R. M. Ryan , 1st ed., 3–C1P190. Oxford University Press.

[sjop70025-bib-0036] Schulte‐Braucks, J. , A. Baethge , C. Dormann , and T. Vahle‐Hinz . 2019. “Get Even and Feel Good? Moderating Effects of Justice Sensitivity and Counterproductive Work Behavior on the Relationship Between Illegitimate Tasks and Self‐Esteem.” Journal of Occupational Health Psychology 24, no. 2: 241–255. 10.1037/ocp0000112. 29683712

[sjop70025-bib-0037] Semmer, N. K. , N. Jacobshagen , L. L. Meier , et al. 2015. “Illegitimate Tasks as a Source of Work Stress.” Work & Stress 29, no. 1: 32–56. 10.1080/02678373.2014.1003996. 25892839 PMC4396521

[sjop70025-bib-0038] Semmer, N. K. , F. Tschan , N. Jacobshagen , et al. 2019. “Stress as Offense to Self: A Promising Approach Comes of Age.” Occupational Health Science 3, no. 3: 205–238. 10.1007/s41542-019-00041-5. 32647746 PMC7328775

[sjop70025-bib-0039] Semmer, N. K. , F. Tschan , L. L. Meier , S. Facchin , and N. Jacobshagen . 2010. “Illegitimate Tasks and Counterproductive Work Behavior.” Applied Psychology 59, no. 1: 70–96. 10.1111/j.1464-0597.2009.00416.x.

[sjop70025-bib-0040] Slemp, G. R. , M. L. Kern , K. J. Patrick , and R. M. Ryan . 2018. “Leader Autonomy Support in the Workplace: A Meta‐Analytic Review.” Motivation and Emotion 42, no. 5: 706–724. 10.1007/s11031-018-9698-y. 30237648 PMC6133074

[sjop70025-bib-0041] Trépanier, S.‐G. , C. Peterson , M. Gagné , C. Fernet , J. Levesque‐Côté , and J. L. Howard . 2023. “Revisiting the Multidimensional Work Motivation Scale (MWMS).” European Journal of Work and Organizational Psychology 32, no. 2: 157–172. 10.1080/1359432X.2022.2116315.

[sjop70025-bib-0047] Van den Broeck, A. , D. L. Ferris , C.‐H. Chang , and C. C. Rosen . 2016. “A Review of Self‐Determination Theory's Basic Psychological Needs at Work.” Journal of Management 42, no. 5: 1195–1229. 10.1177/0149206316632058.

[sjop70025-bib-0042] Van den Broeck, A. , J. L. Howard , Y. Van Vaerenbergh , H. Leroy , and M. Gagné . 2021. “Beyond Intrinsic and Extrinsic Motivation: A Meta‐Analysis on Self‐Determination Theory's Multidimensional Conceptualization of Work Motivation.” Organizational Psychology Review 11, no. 3: 240–273. 10.1177/20413866211006173.

[sjop70025-bib-0043] Vansteenkiste, M. , B. Soenens , and R. M. Ryan . 2023. “Basic Psychological Needs Theory: A Conceptual and Empirical Review of Key Criteria.” In The Oxford Handbook of Self‐Determination Theory, edited by R. M. Ryan , 1st ed., 84–C4P292. Oxford University Press. 10.1093/oxfordhb/9780197600047.013.5.

[sjop70025-bib-0044] Warr, P. , J. Cook , and T. Wall . 1979. “Scales for the Measurement of Some Work Attitudes and Aspects of Psychological Well‐Being.” Journal of Occupational Psychology 52, no. 2: 129–148. 10.1111/j.2044-8325.1979.tb00448.x.

[sjop70025-bib-0045] Zeng, X. , Y. Huang , S. Zhao , and L. Zeng . 2021. “Illegitimate Tasks and Employees' Turnover Intention: A Serial Mediation Model.” Frontiers in Psychology 12: 739593. 10.3389/fpsyg.2021.739593. 34777127 PMC8580953

[sjop70025-bib-0046] Zhao, L. , L. W. Lam , J. N. Y. Zhu , and S. Zhao . 2022. “Doing It Purposely? Mediation of Moral Disengagement in the Relationship Between Illegitimate Tasks and Counterproductive Work Behavior.” Journal of Business Ethics 179, no. 3: 733–747. 10.1007/s10551-021-04848-7.

